# Hospital physicians’ and older patients’ agreement with individualised STOPP/START-based medication optimisation recommendations in a clinical trial setting

**DOI:** 10.1007/s41999-022-00633-5

**Published:** 2022-03-15

**Authors:** C. J. A. Huibers, B. T. G. M. Sallevelt, J. M. J. op Heij, D. O’Mahony, N. Rodondi, O. Dalleur, R. J. van Marum, A. C. G. Egberts, I. Wilting, W. Knol

**Affiliations:** 1 Geriatric Medicine Department, University Medical Center Utrecht, Utrecht, The Netherlands; 2 Clinical Pharmacy Department, University Medical Center Utrecht, Utrecht, The Netherlands; 3 Department of Medicine (Geriatrics), University College Cork and Cork University Hospital, Cork, Ireland; 4 Institute of Primary Health Care (BIHAM), University of Bern, Bern, Switzerland; 5 Department of General Internal Medicine, Inselspital, Bern University Hospital, University of Bern, Bern, Switzerland; 6 Pharmacy Cliniques Universitaires Saint-Luc, Université catholique de Louvain, Louvain, Belgium; 7 Louvain Drug Research Institute-Clinical Pharmacy, Université catholique de Louvain, Louvain, Belgium; 8 Department of Elderly Care Medicine, Amsterdam, UMC, Location VUmc, Amsterdam, The Netherlands; 9 Departments of Geriatrics and Clinical Pharmacology, Jeroen Bosch Hospital, ‘s-Hertogenbosch, The Netherlands; 10 Division of Pharmacoepidemiology and Clinical Pharmacology, Utrecht University, Utrecht, The Netherlands

**Keywords:** Pharmacotherapy optimisation, STOPP/START criteria, Shared-decision-making CDSS, Polypharmacy, Multimorbidity

## Abstract

**Objective:**

To evaluate the agreement of hospital physicians and older patients with individualised STOPP/START-based medication optimisation recommendations from a pharmacotherapy team.

**Methods:**

This study was embedded within a large European, multicentre, cluster randomised controlled trial examining the effect of a structured medication review on drug-related hospital admissions in multimorbid (≥ 3 chronic conditions) older people (≥ 70 years) with polypharmacy (≥ 5 chronic medications), called OPERAM. Data from the Dutch intervention arm of this trial were used for this study. Medication review was performed jointly by a physician and pharmacist (i.e. pharmacotherapy team) supported by a Clinical Decision Support System with integrated STOPP/START criteria. Individualised STOPP/START-based medication optimisation recommendations were discussed with patients and attending hospital physicians.

**Results:**

139 patients were included, mean (SD) age 78.3 (5.1) years, 47% male and median (IQR) number of medications at admission 11 (9–14). In total, 371 recommendations were discussed with patients and physicians, overall agreement was 61.6% for STOPP and 60.7% for START recommendations. Highest agreement was found for initiation of osteoporosis agents and discontinuation of proton pump inhibitors (both 74%). Factors associated with higher agreement in multivariate analysis were: female gender (+ 17.1% [3.7; 30.4]), ≥ 1 falls in the past year (+ 15.0% [1.5; 28.5]) and renal impairment i.e. eGFR 30–50 ml/min/1.73 m^2^; (+ 18.0% [2.0; 34.0]). The main reason for disagreement (40%) was patients’ reluctance to discontinue or initiate medication.

**Conclusion:**

Better patient and physician education regarding the benefit/risk balance of pharmacotherapy, in addition to more precise and up-to-date medical records to avoid irrelevant recommendations, will likely result in higher adherence with future pharmacotherapy optimisation recommendations.

**Clinical trial registration:**

Trial Registration Number NCT02986425.

## Key Summary Points

**Aim:**

To evaluate the agreement of hospital physicians and older patients with individualised STOPP/START based medication optimisation recommendations from a pharmacotherapy team.

**Findings:**

In total, 371 recommendations were discussed with patients and physicians, overall agreement was 61.6% for STOPP and 60.7% for START recommendations. Highest agreement (74%) was found for initiation of osteoporosis agents and discontinuation of proton pump inhibitors.

**Message:**

Better patient and physician education regarding the benefit/risk balance of pharmacotherapy, in addition to more precise and up-to-date medical records to avoid irrelevant recommendations, will likely result in higher adherence with future pharmacotherapy optimisation recommendations.

## Background

Multimorbidity and polypharmacy remain challenging in the context of rapidly ageing populations globally. Although polypharmacy is often indicated in older patients with multimorbidity, it is also associated with an increased risk of negative health outcomes including adverse drug reactions (ADRs) and drug-related hospital admissions (DRAs) [1–3]. Periodic evaluation of the individual patient’s pharmacotherapy by medication review is important to ensure an optimised balance between therapeutic and preventive benefits and potential harms of treatment [4–6].

Several screening tools, both implicit and explicit, have been developed to assist physicians and pharmacists in performing medication reviews [7]. The STOPP/START criteria are explicit criteria that are widely used in medication reviews for older people, especially in Europe [8, 9]. It can, however, be challenging to translate the general population-based STOPP/START recommendations into specific recommendations for the individual patient. An important element of medication review is the alignment of a patient’s pharmacotherapy with individual patient’s preferences [10]. Prior research shows that taking patients’ preferences into account will likely result in higher agreement with recommendations [11–13]. Prescriber implementation of pharmacotherapy optimisation recommendations provided by physicians or pharmacists showed large variation in previous studies [14]. Therefore, it is important to investigate the factors that influence the willingness of patients and their attending physicians to follow pharmacotherapy optimisation recommendations and to understand patients’ and physicians’ reasons for disagreement with the recommendations. This could help to improve the effectiveness of medication reviews, increase appropriate prescribing and ultimately reduce negative health outcomes.

The aim of the current study was to evaluate the level of agreement, including reasons for disagreement, of hospital physicians and older patients with polypharmacy and multimorbidity with individualised STOPP/START-based medication optimisation recommendations from a pharmacotherapy team.

## Methods

### Setting, design and study population

This study was embedded within The Optimising thERapy to prevent Avoidable hospital admissions in Multimorbid older people (OPERAM) clinical trial [15]. In brief, OPERAM was a large European, multicentre, cluster randomised controlled trial examining the effect of a structured medication review on drug-related hospital admissions (DRAs) in multimorbid (≥ 3 chronic conditions) older people (≥ 70 years) with polypharmacy (≥ 5 chronic medications). In-hospital patients were recruited in Switzerland (Bern), Belgium (Louvain), Ireland (Cork) and the Netherlands (Utrecht) i.e. one centre per country. All patients were admitted to the participating hospitals, either electively or non-electively through the emergency department and were recruited in both surgical and medical wards. Geriatric specialist wards were excluded from the OPERAM trial to avoid contamination of the trial arising from routine medication reconciliation and optimisation in such wards. Only data from the Dutch intervention patients were eligible for the present study, as data regarding the agreement with the recommendations and reasons for disagreement by both patients and physicians were only systematically collected at the St. Antonius Hospital, a large non-academic teaching hospital, located in Utrecht and Nieuwegein. Data were collected between January 2017 and October 2018 during the recruitment phase of the OPERAM trial. Baseline characteristics were registered in and extracted from the electronic Case Report Form (eCRF) deployed in each randomised patient.

### Intervention

The intervention within the OPERAM trial consisted of a structured medication review based on the software-supported Systematic Tool to Reduce Inappropriate Prescribing (STRIP) method performed by a pharmacotherapy team (PT), consisting of a physician and a pharmacist, both experienced with geriatric pharmacotherapy optimisation and trained by standardised operating procedures in all trial sites [7, 16]. The Dutch PT consisted of one physician/pharmacist pair performing the intervention throughout the trial. The intervention consisted of five consecutive steps and occurred within 72 h after trial enrolment: (1) Structured History taking of Medication use (SHiM) [17] and collection of patient data including medical conditions, laboratory data and clinical parameters; (2) digitalized screening of pharmacotherapy supported by a Clinical Decision Support System (CDSS) with integrated STOPP/START criteria (version 2) [18, 19]; START and STOPP signals generated by the CDSS were based on the patient data and current pharmacotherapy; (3) pharmacotherapy analysis resulted in a report with individualised recommendations: the CDSS-generated STOPP/START signals were assessed for appropriateness for the individual patient by the PT based on additional information from the patient’s medical records, such as prior use and effectiveness, side-effects or known drug allergies; (4) discussion of individualised medication optimisation recommendations with the patient and attending physician by the PT. Recommendations were first discussed with the patient. The recommendations agreed upon by the patient were then suggested to the attending physician. In case the attending physician did not agree or did not feel qualified to adjust the medication, these recommendations were then transferred to the GP in case both the attending physician and the patient consented; (5) an overview of the recommendations (both implemented during hospital admission and postponed) was transferred to the patient’s GP as a written advice report. The GP was asked to review the postponed recommendations for implementation after hospital discharge in collaboration with the patient.

**Fig. 1:**
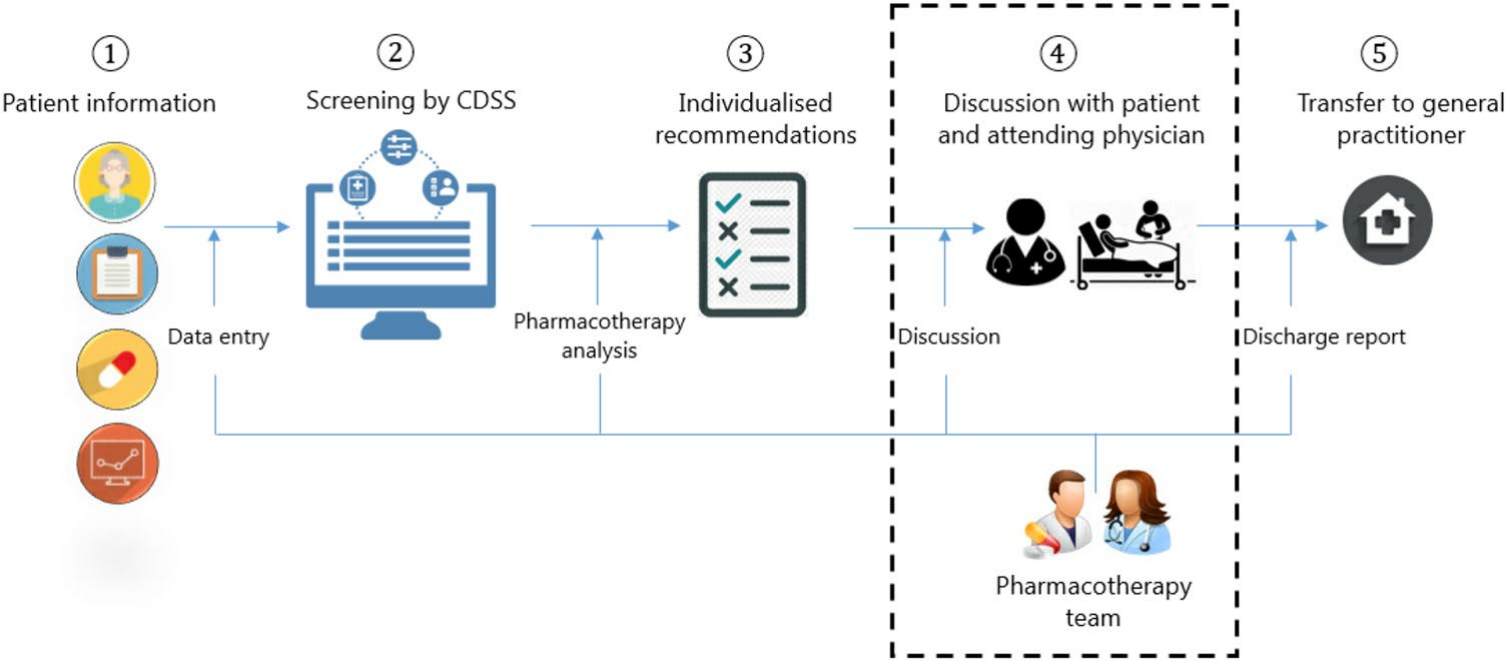
Summary of all consecutive steps (1–5) of the intervention within the OPERAM trial and the focus of this study highlighted: the agreement of recommendations by patients and attending physicians after discussion with the pharmacotherapy team (step 4)

All consecutive steps and the focus of this study (step 4) are summarised in Fig. 1.

### Ethics approval

The local ethics committee at each participating trial site approved the OPERAM study protocol, registered under Trial Registration Number NCT02986425. No additional ethical approval was needed for this study, as the data collected and analysed were part of the main trial [20].

### Primary outcome

The primary outcome of this study was defined as the STOPP/START recommendations provided by the PT that were agreed upon by both patient and the attending hospital physician after discussion with the PT, as illustrated in Fig. 1 (step 4).

### Secondary outcome

Reasons for disagreement with the STOPP/START recommendations by the patient and/or attending hospital physician were collected and analysed.

### Determinants

Potential determinants of agreement with the recommendations were investigated. Potential determinants with continuous values were dichotomised or categorised into tertiles based on the patient distribution (age, comorbidities, number of medications) or based on clinically accepted cut-off values for measurements (renal function). STOPP/START criteria-related variables were: type of recommendation (STOPP versus START), medication involved (i.e. drug class) and a number of recommendations per patient. Patient-related variables include sex, age group (70–79 years, 80–89 years, ≥ 90 years), number of comorbidities (< 7, 7–9 or ≥ 9), renal function (eGFR < 30, 30–50 or ≥ 50 ml/min/1.73 m^2^), the occurrence of falls in the past year (defined categorically as 0 or ≥ 1), and number of long term daily medications at inclusion (< 9, 9–12 or ≥ 12). Setting-related variables were: ward type (medical or surgical) and hospital length of stay (< 7, 7–14, > 14 days).

### Data analyses

Data analysis was performed with IBM SPSS^®^ Statistics v.25.0.0.2. Baseline characteristics and agreement with STOPP/START recommendations were analysed using descriptive statistics. The outcome agreement was binary on a recommendation level (yes/no) and continuous on an individual patient level (percentage of recommendations agreed upon), as multiple recommendations could be applicable to one patient. Potential determinants of the agreement were investigated on an individual patient level using a univariate and multivariate linear regression model (method: enter). For subgroup analyses on a recommendation level, relative risks (RR) and 95% confidence intervals (CIs) were calculated. *P* values < 0.05 were considered statistically significant.

## Results

### Study population

**Fig. 2:**
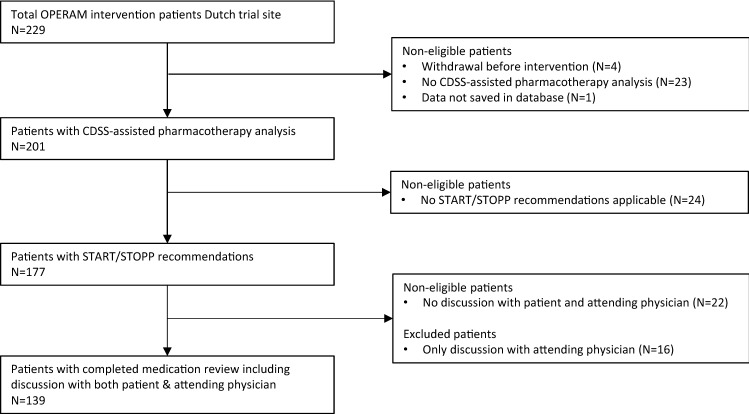
Study population flowchart. Non-eligible patients did not fulfill the inclusion criteria of this OPERAM substudy i.e. discussion of recommendations with patient and attending physician to determine agreement with recommendations

A total of 452 patients were included in the OPERAM cohort at the Utrecht trial site, of whom 229 (50.7%) were allocated to the intervention group. Four patients (1.7%) withdrew from the trial prior to the intervention. The medication review including CDSS-assisted pharmacotherapy analysis was not completed in 23 of 225 patients (10.2%) due to several (mostly logistic) factors, such as early discharge, transfer to another ward (including the Intensive Care Unit) or to another hospital. Data from one patient were missing from the database. In 24 patients, the pharmacotherapy analysis did not result in START/STOPP recommendations. In 22 patients, discussion with patient and physician was not performed and for 16 patients recommendations were only discussed with the attending physicians and not with the patients. These 16 patients were excluded from the final analysis. For 139 of the 155 eligible patients (89.7%), the medication review including discussion with both patient and attending physician was successfully completed. These 139 patients comprised the study population. A flowchart illustrating the data flow is presented in Fig. 2.

The mean (SD) age of the study population was 78.3 (5.1) years, 65 patients (47%) were male and the median (IQR) number of prescribed long-term daily medications prior to admission was 11 (9–14). All baseline characteristics are presented in Table 1.

**Table 1:** Baseline characteristics of the study population

Characteristics	
Patients, *N*	139
Age in years, mean (SD)	78.3 (5.1)
Gender (Male), *N* (%)	66 (47.5%)
Number of comorbidities, median (IQR)	8 (6–11)
Number of prescribed medications (admission), median (IQR)	11 (9–14)
Nursing home residents, *N* (%)	6 (4.3%)
Housebound patients, *N* (%)	19 (13.7%)
Barthel Index of ADL, median (IQR)	92.5 (85–100)
Patients with ≥ 1 fall(s) in the past year, *N* (%)	57 (41.9%)
Patients with ≥ 1 hospital admission in the past year, %	67 (48.2%)
Length of stay index hospitalisation in days, median (IQR)	9 (6–18)
Estimated GFR (CKD-EPI, mL/min/1.73 m^2^) Mean (SD)	59.1 (20.6)
Estimated GFR 30–50 ml/min/1.73 m^2^, *N* (%)	36 (25.9%)
Estimated GFR ≤ 30 ml/min/1.73 m^2^, *N* (%)	13 (9.4%)
Ward (*N*, %)	
Medical	109 (78.4)
Surgical	30 (21.6)
Admission type (*N*, %)	
Elective	34 (24.5)
Non-elective	105 (75.5)

Missing data: number of comorbidities 3 (2.2%) renal function 5 (3.6%) nursing home residents & housebound 1 (0.7%) Barthel Index 1 (0.7%) Falls 3 (2.2%) hospitalisations 1 (0.7%)

CDSS-assisted pharmacotherapy analysis by the PT resulted in a total of 371 recommendations for 139 patients, comprising 237 STOPP recommendations (median (IQR): 1 (1–2) per patient) and 134 START (1 (0–1) per patient) recommendations. Overall STOPP/START recommendation agreement was 61.2%, with no significant difference in agreement proportion between STOPP (61.6%) and START (60.7%) recommendations.

### Agreement with recommendations based on STOPP criteria

Among all 237 STOPP recommendations discussed, 146 (61.6%) were agreed upon by both patient and physician. More than half (52.7%) of the STOPP recommendations discussed with the patients and physicians were based on criterion ‘no evidence-based clinical indication’ (STOPP A1), of which there was consensus to discontinue in 60.8% after discussion.

Within the STOPP A1 criterion (‘no evidence-based clinical indication’), drugs for acid-related disorders (including PPIs) represented 43.2% of the recommendations. After discussion with both patient and attending physician, 74.1% of these recommendations relating to drugs for acid-related disorders were agreed upon. Other medication groups within STOPP A1 were heterogeneous and contained small numbers with varying agreement e.g. inhaled bronchodilators (*N* = 12; 33.3% agreement), analgesics (*N* = 7; agreement 28.6%).

**Fig. 3:**
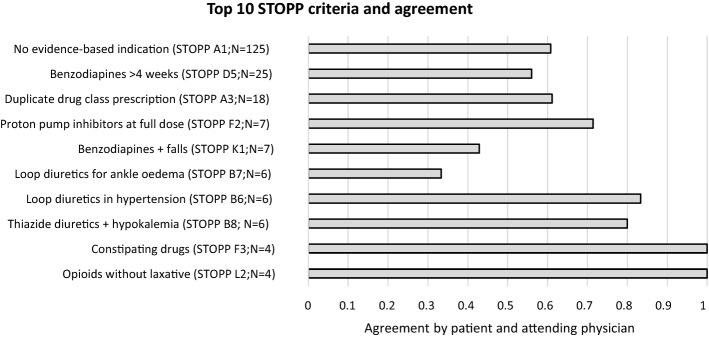
Top 10 STOPP recommendations and corresponding agreement by patient and attending physician after discussion with PT. STOPP A1: ‘No evidence-based clinical indication’ contains stop recommendations for multiple medications with ‘drugs for acid-related disorders’ being the most prevalent (43.2% of STOPP A1)

The 10 most prevalent STOPP recommendations, comprising 87.3% (*N* = 207) of all discussed STOPP recommendations and their subsequent agreement by both patient and attending physician after discussion with PT are listed in Fig. 3. Some of these individual criteria contain STOPP recommendations for the same medication (or drug class) but were based on other reasons for inappropriateness. For example, implementing STOPP criteria D5 and K1 both result in discontinuation advice for benzodiazepines.

### Agreement with recommendations based on START criteria

**Fig. 4:**
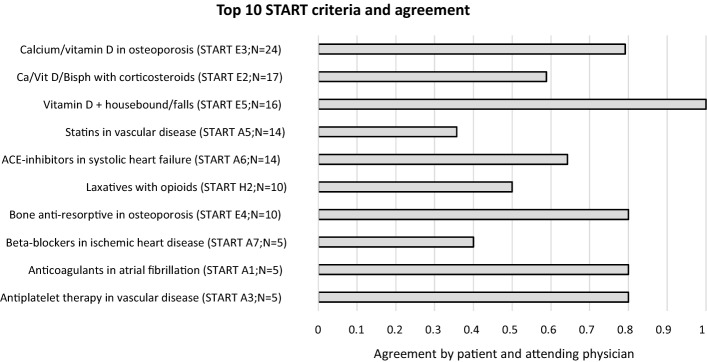
Top 10 START recommendations and corresponding agreement by the patient and attending physician after discussion with PT. START E3 consists of recommendations for both calcium and/or vitamin D. START E2 consists of recommendations for calcium, vitamin D and/or bisphosphonates (i.e. Ca/Vit D/Bisph in the figure)

Of the 134 START criteria discussed with patients and their attending physicians by the PT, 60.7% were agreed upon. An overview of the 10 most prevalent START recommendations, comprising 89.6% (*N* = 120) of all START recommendations discussed and subsequent agreement, is displayed in Fig. 4.

### Determinants of agreement

Potential determinants of the agreement were investigated on a patient level (*N* = 139). Multivariate linear regression revealed three patient-related factors significantly associated with higher mean agreement (with STOPP/START recommendations taken together) i.e. female gender (+ 17.1% [3.7; 30.4]), ≥ 1 falls in the past year (+ 15.0% [1.5; 28.5]) and moderately diminished renal function defined as eGFR 30–50 ml/min/1.73 m^2^ (+ 18.0% [2.0;34.0]). None of the investigated setting-related factors (ward type, admission type, length of stay) was associated with lower/higher agreement. All determinants included in the univariate and multivariate analyses are displayed in Table 2.

**Table 2:** Statistical analysis of determinants of agreement

Determinant	Patients (*N*)	Mean agreement (%)	Linear regression (% [95%-CI])	
Patient related determinants			Univariate	Multivariate
Gender				
Male	66	52.9	Ref	Ref
Female	73	68.7	** + 15.8 [3.2; 28.4]**	** + 17.1 [3.7; 30.4]**
Age				
< 75	43	62.2	Ref	Ref
75–80	45	56.6	− 5.7 [− 21.7; 10.4]	− 3.9 [− 19.9; 12.1]
> 80	51	64.3	+ 2.0 [− 13.7; 17.6]	− 2.4 [− 18.8; 14.1]
*Number of co-morbidities*				
< 7	38	63.1	Ref	Ref
7–9	52	59.8	− 3.3 [− 19.5; 12.9]	− 6.8 [− 23.6; 9.9]
> 9	49	61.2	− 1.9 [− 18.3; 14.5]	− 3.4 [− 21.1; 14.4]
Number of medications				
< 9	34	57.4	Ref	Ref
9–12	54	61.2	+ 3.8 [− 12.8; 20.4]	− 7.7 [− 24.6; 9.3]
> 12	51	63.7	+ 5.52 [− 11.42; 22.45]	− 8.1 [− 25.9; 9.7]
Number of falls in the past year				
0	79	55.1	Ref	Ref
≥ 1	57	69.3	** + 14.1 [1.3; 27.0]**	** + 15.0 [1.5; 28.5]**
Number of hospital admissions in the past year				
0	70	65.0	Ref	Ref
≥ 1	68	56.7	− 8.3 [− 21.1; 4.5]	− 6.1 [− 19.2; 7.0]
Renal function (eGFR; CKD-EPI; ml/min/1.73 m^2^)				
> 50	86	57.8	Ref	Ref
30–50	37	72.9	** + 15.1 [0.5; 29.8]**	** + 18.0 [2.0; 34.0]**
< 30	13	53.0	− 4.8 [− 27.0; 17.4]	− 6.3 [− 29.6; 17.1]
Setting related determinants				
Ward				
Medical	109	60.0	Ref	
Surgical	30	65.3	+ 5.3 [− 10.3; 20.9]	
Admission type				
Elective	34	60.1	Ref	
Non-elective	105	61.5	+ 1.4 [− 13.5; 16.4]	
Length of stay (days)				
< 7	38	57.0	Ref	
7–14	58	60.6	+ 3.6 [− 12.2; 19.4]	
> 14	43	65.7	+ 8.7 [− 8.2; 25.5]	

All patient and setting-related determinants were included in the univariate linear regression model. Determinants significantly associated with the higher agreement were included in the multivariate model (cut-off value *p* < 0.2)

Other variables of interest (age, number of comorbities and number of medications) were also included in the multivariate analysis. All values including 95% confidence intervals are shown. Statistically significant values are in bold

*Ref* reference category

**Fig. 5:**
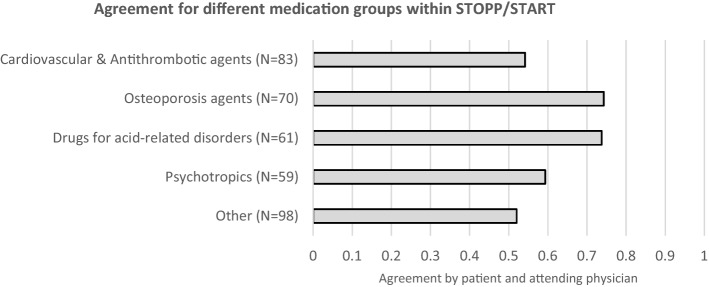
Categorisation of individual STOPP/START recommendations (N = 371) into 5 medication groups and subsequent agreement after discussion with the patient and attending physician. Note: Groups ‘psychotropics’ and ‘drugs for acid related disorders’ contain only STOPP recommendations, ‘osteoporosis agents’ 3 STOPP and 67 START, ‘cardiovascular & antithrombotic agents’ 35 STOPP and 48 START and the group ‘other’ contained 79 STOPP and 19 START recommendations

For the individual STOPP and START recommendations (*N* = 371), potential determinants of the agreement were investigated as well. No difference was found between STOPP and START recommendations and no significant relationship was found between the number of recommendations discussed (range 1–7) and subsequent agreement. All individual STOPP and START recommendations were categorised into subgroups according to the medication class involved and their occurrence. This resulted in 4 subgroups: (1) cardiovascular & antithrombotic agents (*N* = 83; 22.4%), 2) drugs for acid related disorders (*N* = 61;16.4%), psychotropic drugs including benzodiazepines/Z-drugs (*N* = 59; 15.9%), (3) osteoporosis agents (vitamin D, calcium and bisphosphonates; *N* = 70;18.9%) and (4) miscellaneous others (all other medications, *N* = 98;26.4%). The levels of agreement with PT recommendations within these groups is displayed in Fig. 5. Within these medication groups, agreement varied when stratified for gender, with significantly higher agreement in females for cardiovascular medications, i.e. 66.7% versus 41.5% by males (RR 1.61; 95%CI 1.05–2.45; *p* = 0.0274) and osteoporosis drugs, i.e. 91.9% versus 54.5% (RR 1.68; 95%CI 1.21–2.33; *p* = 0.0017). A history of ≥ 1 falls in the previous year resulted in a significantly higher agreement with recommendations regarding osteoporosis drugs i.e. 94.6% versus 51.5% among patients with no falls (RR 1.84; 95%CI 1.31–2.58; *p* = 0.0005).

### Reasons for disagreement with recommendations

From the total of 371 STOPP/START recommendations that were discussed with both patient and attending physician, 143 (38.5%) were not agreed upon with ‘patient does not agree’ being the most prevalent documented reason for disagreement (39.9%).

The majority of recommendations to discontinue *drugs for acid-related disorders* (*N* = 61; of which 95.1% involved PPIs) were agreed upon (73.8%, Fig. 5). Disagreement within this drug class occurred in 31% due to reluctance to discontinue by the patient, mainly relating to previous ineffective attempts to discontinue the medication. In another 31% of recommendations, the medication adjustment decision was deferred to the patient’s GP. In 19% of recommendations, they were no longer applicable at the time of discussion, indicating that new information had emerged during the discussion that was not present in the patient’s medical records. The remaining 19% of non-agreed recommendations were defined as ‘other’ or ‘unknown’ reasons.

Within the psychotropic medication group, 49 recommendations involved stopping benzodiazepines or Z-drugs. Of these, 27 recommendations (55.1%) were agreed upon by both patient and physician. Disagreement, when it occurred, was in the great majority (90.9%) due to reluctance to discontinue by the patient. The most common reasons given were chronic use without side effects (falls or sleepiness) and self-reported dependence by patients.

Recommendations to start osteoporosis drugs (*N* = 67) were agreed upon by both patient and physician in 74.3% of cases. Reasons for disagreement included recommendation no longer applicable (41%) based on new information obtained during a discussion with patient/physician, patient not agreeing (35%) based on lack of motivation to take more tablets, and patient preference to discuss the matter with their GP rather than stopping in hospital. For 12 recommendations (18%), the decision was deferred to the GP and in the remaining 4 recommendations (6%), the reason for disagreement was unknown.

Medication within the cardiovascular and antithrombotic agents group contained both START recommendations (*N* = 48) and STOPP recommendations (*N* = 35) with identical mean levels of agreement for both categories, i.e. 54%. In cases of disagreement, the most important reason was ‘physician does not agree or does not feel qualified to advise’ (30%). In 24% of recommendations, the decision was deferred to the GP. In 19% of recommendations, the reason was ‘patient does not agree’. In 5%, the recommendation was no longer applicable and in 22% other reasons were applicable or the reason was not known.

## Discussion

In this study we evaluated older patients’ and their attending hospital physicians’ agreement/disagreement with individualised STOPP/START criteria-based medication optimisation recommendations from a pharmacotherapy team. Overall agreement was 61.6% for STOPP recommendations and 60.7% for START recommendations, after discussion of 371 recommendations with 139 patients and their attending physicians. The most frequently discussed recommendation was ‘no evidence-based clinical indication’ (STOPP A1;33.7% of all recommendations). Highest agreement was found for initiation of osteoporosis agents and discontinuation of drugs for acid-related disorders (both 74%).

Few studies have explored patients’ or physicians’ agreement with in-hospital pharmacotherapy optimisation recommendations. In a non-randomised study among older patients admitted to a specialist geriatric unit, physicians’ agreements with STOPP recommendations, including benzodiazepines, was 87% compared to 62% in our study, presumably explained by the lack of patient involvement in decision making in contrast to our study [21]. Reasons for disagreement with STOPP/START recommendations in that study were predominantly ‘therapeutic prioritisation’ (STOPP) and ‘severe mental or physical disability’ (START). Differences may be explained by a different study population (mean age 88.5, high prevalence of severe dementia (32%) and high prevalence of severe ADL deficiencies (50%)) compared to our study [21].

In the present study, reasons for disagreement varied between medication groups. Disagreement with the stopping of benzodiazepines and Z-drugs was, in 90.9% of instances, due to reluctance to discontinue by the patient (e.g. self-reported dependence, lack of side effects). Low perceived necessity to discontinue medication, as with benzodiazepines in our study, acted as a barrier to an agreement with in-hospital medication changes in a qualitative study among older polypharmacy patients [22]. Conversely, the majority of these patients reported acceptance of the hospital-initiated medication changes with high perceived importance (e.g. usual treatment ineffective or causing side-effects). This could explain our findings that initiation of osteoporosis drugs in patients who experienced a fall in the previous year had significantly higher agreement than in patients with no falls (94.6% versus 51.5%).

Research shows that many patients expressed the wish to reduce their daily number of medications [22]. However, patients’ willingness to deprescribe specific medications, like benzodiazepines/Z-drugs, was considerably lower in our study than the hypothetical willingness to discontinue medication reported by other researchers (around 90%), investigating patients’ attitudes, beliefs and willingness related to medication deprescribing through questionnaires [12, 23]. This might partly be explained by the hospital setting in the present study. In addition, potentially inappropriate medication (PIM) use was not associated with patients’ willingness to deprescribe one or more of their medications (74.3% without PIMs versus 79.9% with PIMs) in prior studies [24]. Female gender was associated with more PIM use (based on Beers criteria), especially benzodiazepines, Z-drugs and ≥ 3 concurrent psychoactive drugs, but not with a willingness to deprescribe. We found no gender difference in PIM or PPO prevalence, but we did find an association between female gender and higher agreement with recommendations (both STOPP and START). This is an interesting new finding that needs to be confirmed in future research.

Although patients’ reluctance to medication adjustments was an important reason for disagreement, factors within the attending physician and environmental constraints were also prevalent. Postponed recommendations to the GP (21% in total) were frequently associated with attending physicians feeling ill-equipped to take responsibility for suggested medication changes beyond their area of expertise, as we found for cardiovascular medication. These factors correspond relatively well with those found by Dalton et al., who investigated factors affecting prescriber implementation of computer-generated medication recommendations within the SENATOR trial [25, 26]. Although the SENATOR-derived study significantly differs in methodology and outcome from our study, four important barriers for implementation were elucidated, of which some were partly overcome in our trial, i.e. (1) computerised output leading to recommendations with low clinical relevance, thereby limiting their uptake; (2) the hospital environment with associated time constraints within the busy clinical environment and desire to devolve the responsibility of managing older patients’ pharmacotherapy to GPs; (3) prescriber factors, particularly prescriber inertia and lack of awareness of the highly prevalent ADRs, reluctance to prescribe outside their therapeutic specialty; (4) patient factors, particularly the overriding focus on the patient’s acute status, where reviewing the prescribing recommendations was not a high priority for many attending physicians [25]. All pharmacotherapy optimisation recommendations that were discussed with the patient and the physician in our study, were already evaluated for appropriateness for the individual patient by the PT. This resulted in the rejection of 603 out of 1059 (56.9%) STOPP/START signals generated by the CDSS during pharmacotherapy analysis in Dutch patients, based on information present in the patients’ medical records (results of this evaluation process are published elsewhere) [16, 27]. Therefore, the category ‘computerised output’ was not applicable to our study, as all recommendations discussed were considered relevant to the patient by the PT. Additionally, our output was discussed face-to-face with both patient and attending physician, in contrast to providing a printed report with recommendations to the attending physician and nothing more. These factors would likely contribute to higher implementation rates than those found in the SENATOR trial (15%) and could explain the overall agreement of 60% we found in our study [26]. In the OPERAM main trial, at least one of the recommendations was successfully implemented at 2 months follow-up in 62.2% of the patients who received ≥ 1 recommendation during the intervention (across all participating countries). This primarily concerned the discontinuation of potentially inappropriate medications (STOPP A1) and duplicate drug class prescriptions (STOPP A3) [28]. Interestingly, the recommendation by PTs to discontinue benzodiazepines used ≥ 4 weeks (STOPP D5), was implemented in 39.1% at 2 months, suggesting that the majority (80%) of these recommendations agreed upon during discussion (55.1% in our study) were actually implemented after discharge and still discontinued at 2 months. As for START criteria, implementation was considerably lower at 2 months ranging from 12.7% for ‘bone antiresorptive treatment’ in osteoporosis (START E4) to 38.8% for vitamin D supplements in housebound patients (START E5). Although these OPERAM results reflect all participating trial sites and the agreement presented in this study concerns only the Dutch trial site, these numbers confirm our hypothesis that many possible factors impede the actual and persistent implementation of (verbally) agreed upon recommendations after hospital discharge.

### Limitations

This study has some limitations. First, data were collected in a single centre and represent a relatively small sample. Secondly, the entire intervention including CDSS analysis and discussion with both patient and attending hospital physician (in cases where STOPP/START recommendations were applicable), as intended by the OPERAM trial protocol [15], was not completed in 66 of 229 (28.8%) Dutch patients which could have introduced bias to the results. Also, according to the OPERAM protocol, only numbers of diseases and medications, rather than the prevalence of common diseases and medications, are presented at baseline [28]. This might compromise the generalisability of the results. Thirdly, reasons for disagreement were collected by the PT after discussion with patients and attending physicians, thereby possibly introducing bias during documentation of the reasons. In addition, the ‘patient does not agree’ option could also be interpreted as ‘PT failed to convince the patient’ in some cases. Furthermore, agreement with recommendations mentioned in our study was based on ‘oral consent’ to follow the suggested recommendations by both patients and physicians. Although these percentages might considerably change over time, agreement/disagreement was not re-evaluated after discharge. Moreover, actual implementation of the STOPP and START recommendations at hospital discharge was at the discretion of the attending physician and not measured in this OPERAM substudy. It is likely, however, that whilst attending physicians agreed upon medication adjustments verbally, implementation rates were lower due to practical/logistical reasons (e.g. busy clinical practice, pressure to discharge patients once stable, etc.) or patient-related factors like additional changes in medication due to (acute) intercurrent conditions such as sepsis, pain or dehydration. Finally, communication with the GP was solely through a written report with recommendations to consider after discharge (separately from the hospital discharge letter) and could easily have been missed by the GP. It is likely that adherence by GPs to the postponed recommendations could be improved by discussion through follow-up phone calls to explain and motivate the patients’ GPs to implement prescribing recommendations post-discharge.

### Implications

In this study, high willingness among hospitalised multimorbid older patients and their attending physicians to follow pharmacotherapy optimisation recommendations was found, however, some important areas for improvement were also identified. Disagreement with recommendations was related to the patient’s reluctance to change pharmacotherapy in approximately 40% of cases. Better patient education regarding the potential benefits and harms of pharmacotherapy and training of physicians/pharmacists in shared-decision-making (SDM) to more effectively communicate this information to the patient could attribute to better-informed decision-making and possibly higher agreement [29]. More and better education and explanation about the potential benefits of implementing the suggested pharmacotherapy recommendations is also important for the hospital physicians because they felt that some medication groups were beyond their own area of expertise. The discussion with the patient and physician revealed that medical records were not always up to date, making 13% of the recommendations irrelevant at the time of discussion. To increase the specificity of CDSS-assisted medication reviews, it is important that the necessary clinical information in medical records is current and accurate. Low implementation rates of pharmacotherapy optimisation recommendations in clinical trials impedes drawing firm conclusions about the impact of medication reviews on clinical endpoints like readmissions and mortality, as was recently found in the OPERAM trial [26]. In addition, medication reviews should not be performed at a single time point during admission, but need to be repeated after discharge in close collaboration with the GP and community pharmacists, since nearly 50% of patients are unable to recall medication changes implemented in-hospital [22, 30]. The effects of medication adjustments (both positive and negative) should be closely monitored and recommendations continuously evaluated and adjusted when necessary. In addition, discussion of medication changes with older patients during hospital admissions for acute illnesses and corresponding disturbances of homeostasis, may not be the ideal time to optimise long-term pharmacotherapy. Both patients and prescribers often have other priorities and certain medication changes could have detrimental effects in unstable patients. Not surprisingly, the patient’s GP appears to have a particularly strong influence on medication withdrawal (both for and against) [31, 32]. Trials focusing on optimising pharmacotherapy in multimorbid older people conducted in, or in close collaboration with, primary care physicians are needed to assess whether the clinical setting and the health care professional involved have a significant influence on recommendation agreement, implementation, monitoring and prevention of adverse events within this population.

## Conclusion

Hospital physicians’ and older patients’ agreement with individualised STOPP/START-based medication optimisation recommendations after discussion with a pharmacotherapy team was approximately 60%. Highest agreement was found for initiation of osteoporosis drugs and stopping of PPIs. Female gender, history of falls and eGFR 30–50 ml/min/1.73 m^2^ were significantly associated with higher agreement levels with proposed medication adjustments. Patients’ own reluctance to change (40%) was the most important reason for disagreement. Better patient and physician education regarding the benefit/risk balance of pharmacotherapy in addition to more precise and up-to-date medical records will likely result in higher agreement with and implementation of pharmacotherapy optimisation recommendations in the future.

## Data Availability

Data for this study will be made available to others in the scientific community upon request after publication. Data will be made available for scientific purposes for researchers whose proposed use of the data has been approved by a publication committee.
